# The Emotional Burden of Loneliness and its Association with Mental Health Outcomes

**DOI:** 10.1007/s12529-023-10255-1

**Published:** 2024-01-19

**Authors:** Lambros Lazuras, Antonia Ypsilanti, Emma Mullings

**Affiliations:** 1 School of Sport & Exercise Science, University of Lincoln, England, UK; 2 Centre of Behavioural Science and Applied Psychology, Department of Psychology, Sociology & Politics, Sheffield Hallam University, Sheffield, UK; 3 Department of Psychology, Sociology & Politics, Sheffield Hallam University, Sheffield, UK

**Keywords:** Loneliness, Valence, Arousal, Depression, Anxiety

## Abstract

**Background:**

The present study examined, for the first time, the emotional burden of loneliness on dimensions of emotional valence and arousal, and its association with mental health outcomes.

**Method:**

A cross-sectional design was used, and data were collected from 503 adults across the UK with an online survey. Measures included socio-demographic characteristics, self-reported measures of loneliness and social isolation, affective ratings (i.e., valence and arousal) of loneliness experiences, and symptoms of depression and anxiety as mental health outcomes.

**Results:**

The emotional burden of loneliness differed significantly across groups with differing loneliness experiences, and females scored significantly higher in the emotional burden of loneliness than males. The emotional burden of loneliness was associated with both depression and anxiety symptoms, and respectively added 4.7% and 6.2% of the variance, on top of measures of loneliness frequency and social isolation.

**Conclusions:**

Measuring the valence and arousal dimensions of loneliness experiences advances our understanding of loneliness experiences and its association with mental health outcomes. The theoretical, methodological, and practical implications of our study are discussed.

## Introduction

Loneliness, defined as the discrepancy between the desired and actual meaningful social relationships one has, is theoretically and empirically distinguished from social isolation, which reflects one’s richness of and embeddedness within social networks [1]. A large body of research has indicated that loneliness represents an important risk factor for poor physical and mental health [2], is associated with broad-based morbidity and mortality [3], and its effects on public health are comparable to those of smoking and obesity [4]. Unless evidence-informed preventive action is taken, loneliness will continue to negatively impact public health and increase healthcare costs and costs associated with lost productivity [5, 6].

Most of the existing self-reported measures evaluate the frequency of loneliness experiences. For example, the 3rd version of the UCLA Loneliness Scale [7] requires respondents to indicate the frequency of different loneliness experiences (e.g., “*how often do you feel “in tune” with the people around you?*”), and responses are respectively anchored on a frequency rating continuum (e.g., from “never” to “often”). Similarly, the widely used De Jong Gierveld Loneliness Scale [8] includes sets of questions that reflect different loneliness experiences (e.g., “*I experience a general sense of emptiness*”) and are scored on a frequency continuum (e.g., from “none of the time” to “all of the time”). A common assumption that underlies the extant measures of loneliness is that the more frequent the loneliness experiences, the greater the level of loneliness and, consequently, the greater the impact of loneliness on mental and physical health [9].

However, measuring the frequency of loneliness presents only limited information about loneliness experiences and how those experiences may be associated with other health outcomes. In support of this argument, Qualter et al. [9] used an extended measure of loneliness that incorporated the frequency, intensity, and duration of loneliness. They found that the duration of loneliness was an important factor in identifying groups with more severe experiences of loneliness and associated mental and physical health difficulties and called for future research on expanded loneliness measures. One way of further elucidating the intensity of loneliness experiences is by assessing their emotional content (or burden). Research has shown that emotional loneliness, which is subjectively experienced, and objectives measures of social isolation can be differentially associated with mental health outcomes [10, 11]. This view has been further corroborated by research which showed that some loneliness experiences may elicit lower emotional arousal than others [12, 13].

### Valence and Arousal of Emotional Experiences

Evaluations of valence (i.e., pleasantness vs. unpleasantness) and arousal (i.e., low intensity vs. high intensity) represent key components of affective experiences and can be particularly relevant and useful in understanding the emotional burden of loneliness and its association with mental health outcomes. Research has shown that valence and arousal can differentiate between different emotional experiences at a neural level [14, 15], and predict different mental health outcomes [16]. For instance, people with depression process and respond to emotional stimuli different than people without depression, and emotional valence and arousal modulate this process [17]. In the present study, our contention is that loneliness experiences can be evaluated along the dimensions of valence and arousal, inasmuch the same way emotional stimuli are evaluated. Accordingly, valence and arousal ratings of loneliness experiences can be assessed alongside measures of loneliness frequency. Theoretical support for this idea comes from two paradigms. Firstly, the *Affectivism* paradigm suggests that assessments of emotional experiences should incorporate the effects of emotional valence and arousal [e.g., 18]. Secondly, the neuroscience paradigm of loneliness [e.g., 19] suggests that affective processes and the neural networks that modulate them play an important role in regulating loneliness experiences and motivating behaviour, either to socially re-connect or withdraw. In this context, valence and arousal play a key role in perceiving, interpreting, and responding to social interactions and social stimuli [20]. Therefore, integrating valence and arousal assessments in traditional loneliness measures allows for a better understanding of how often a person experiences loneliness (frequency), to what extent are loneliness experiences pleasant or unpleasant (valence), and how emotionally intense (arousal) are those experiences.

To the best of the authors’ knowledge, no previous research has examined the emotional burden of loneliness using affective ratings of valence and arousal. The aim of the present study, therefore, was to assess the emotional burden of loneliness, by incorporating measures of valence and arousal alongside the assessment of the frequency of loneliness experiences, which is typically used to indicate the prevalence of loneliness in the population. Relatedly, it was examined whether the emotional burden of loneliness is associated with mental health outcomes, over and above the effects of loneliness frequency and socio-demographic factors that have been associated with loneliness in previous research [e.g., 21, 22], such as sex and age differences, and objective measures of social isolation as reflected in measures of one’s social network size [10].

## Methods

### Participants

For the purposes of the study and given the COVID restrictions in place at the time of data collection, we used the Online Research Platform Prolific (www.prolific.co) to recruit participants from the general population across the UK nations (England, Scotland, Wales, and Northern Ireland). Research has shown that compared to other well-known online research participation platforms (e.g., Amazon’s MTurk), Prolific yields higher quality data across populations and measures [23, 24] and reliable behavioural estimates even in studies using repeated assessments over time, which tend to be affected by higher attrition rates [25], Overall, 503 participants were recruited, 49.3% (*n* = 248) were males, and the majority (76%) were aged between 25 and 64 years. Most of the participants (80.3%) self-identified as White British, Irish, or other White; 8.6% self-identified as Asian British (including those of Chinese heritage); and 5% self-identified as Black British. The ethnic breakdown of the participants in the present study closely resembles the 2021 Census results for the UK. Because the same data set was used for a different purpose in another study, more details about sampling and participant characteristics are presented elsewhere [26].

### Measures

All the measures used in the present study were in English and they are described as follows.

#### Demographics

For the purposes of the present study, demographic characteristics included sex and age.

#### Frequency of Loneliness

Russell’s [7] 10-item UCLA Loneliness Scale was used to assess the frequency of loneliness (e.g., “*How often do you feel that you lack companionship*”, and “*How often do you feel close to people*”), and responses were anchored on a 4-point continuous scale, from 1 (= never) to 4 (= always). Higher scores indicated greater frequency of loneliness, and the internal consistency reliability was high (Cronbach’s *α* = 0.88).

### Emotional Burden of Loneliness

To assess the emotional burden of loneliness we incorporated measures of valence and arousal for each item of the UCLA Loneliness Scale. Specifically, for each item in the UCLA Loneliness Scale participants reported the pleasantness (valence) and the intensity (arousal) of the loneliness experience described therein. For instance, for the item “*How often do you feel that you lack companionship*” in addition to indicating the frequency of this experience, participants also reported the pleasantness and intensity of this experience. Scores on the affective ratings (valence and arousal) were reported on a 10-point continuous scale, ranging from 0 to 9 using a slider bar. After reverse scoring the positively worded loneliness frequency item, the emotional burden of loneliness was calculated by multiplying the scores of loneliness frequency × valence × arousal, so that higher scores indicated higher emotional burden of loneliness (i.e., more *frequent* and more *emotionally negative* and *intense* loneliness experiences).

The rationale for the multiplicative effect (loneliness frequency × valence × arousal) used in the present study is informed by previous theory on the synergistic effect of affective valence and arousal on social experiences and interactions (including social isolation or exclusion). Specifically, arousal can amplify positively or negatively evaluated social experiences. In turn, this mechanism influences the cognitive and emotional processing of such experiences [20], and research has shown that loneliness differentiated the valence and arousal ratings of social interaction cues (e.g., social bonding pictures; [27]). Furthermore, research has suggested that valence and arousal interaction can explain people’s responses to socially threatening or aversive situations [e.g., 28].

#### Depression

Self-reported symptoms of depression were measured with the PHQ-9 [29]. This measure includes different symptoms of depression as described in the DSM-IV symptom criteria (e.g., “*feeling down, depressed, or hopeless*”; and “*feeling tired or having little energy*”). Participants were asked to report depression symptoms over the past 14 days. Responses were anchored on a 4-point continuous scale from “0” (not at all) to “3” (nearly every day). Internal consistency reliability of the PHQ-9 in the present study was high (Cronbach’s *α* = 0.90).

#### Anxiety

The GAD-7 [30] measure was used to assess self-reported anxiety. This measure includes different anxiety symptoms as reflected in the DSM-IV criteria for Generalised Anxiety Disorder. Participants rated the frequency of anxiety symptoms over the past 14 days (e.g., “*trouble relaxing*”; “*not being able to stop or control worrying*”), and responses were anchored on a 4-point continuous scale (0 = not at all, to 3 = nearly every day). Internal consistency reliability of the GAD-7 in the present study was high (Cronbach’s *α* = 0.92).

#### Objective Measure of Social Isolation

The Lubben Social Network Scale – 6 (LSNS-6) [31] was used as an objective measure of the number of social connections (or the lack thereof), using 6 items on a scale from 0 = none to 5 = nine or more. Participants report how many times they have seen family and friends over the last month and a total score is calculated by adding the scores with a range from 0 to 30. In the present study, internal consistency reliability was high (Cronbach’s *α* = 0.94). The score of the LSNS was used in subsequent analysis as an indicator of objective social isolation.

### Design/Procedure

A cross-sectional design was used, and all self-reported measures were completed online via Qualtrics on an electronic device (mobile phone, tablet, PC) that suited the participants. There were no time limitations, and the survey took approximately 15–20 min to complete. Participants were compensated for their time using the standard pay rates of the platform. The study received ethics approval from Sheffield Hallam University Research Ethics Committee.

### Data Analysis

Pearson’s correlation (*r*) was used to assess the bivariate association between the frequency and emotional burden of loneliness, and symptoms of depression and anxiety. Analysis of frequencies was used to classify participants into loneliness, anxiety, and depression groups using the respective cut-off scores. Non-parametric tests (Kruskal-Wallis) were used to assess group differences (between anxiety, depression, and age groups) in the frequency and emotional burden of loneliness. Independent samples t-tests were used to assess sex differences in the emotional burden of loneliness, depression and anxiety symptoms. Finally, hierarchical linear regression analysis was used to assess the multivariate associations between the emotional burden of loneliness and depression and anxiety symptoms, after controlling for loneliness frequency, socio-demographic variables, and social isolation (as reflected in the scores in the social network measure).

## Results

### Association of the Emotional Burden of Loneliness with Loneliness Frequency and Mental Health Outcomes

Bivariate associations between the emotional burden of loneliness and scores of loneliness frequency, social isolation, and mental health symptoms is presented in Table 1. The observed correlations were in the expected direction. Specifically, the emotional burden of loneliness was associated positively with loneliness frequency, depression and anxiety symptoms, and negatively with social network size.

**Table 1:** Associations of the frequency and emotional burden of loneliness with social isolation and mental health outcomes

	1	2	3	4	5
1. Emotional burden of loneliness	-	0.80	− 0.38	0.52	0.49
2. Frequency of loneliness		-	− 0.49	0.48	0.41
3. Social isolation			-	− 0.25	− 0.19
4. Depression symptoms				-	0.68
5. Anxiety symptoms					-
*M*	653.65	22.25	19.46	2.16	1.73
*SD*	462.58	5.51	5.84	3.54	3.24
Cronbach’s *α*	-	0.87	0.84	0.87	0.91

All correlations are significant at *p* < .001

### Loneliness Frequency, Depression, and Anxiety Symptoms

The frequency of loneliness was assessed with the UCLA Loneliness Scale. To determine loneliness groups based on the UCLA Loneliness Scale scores, we applied Russell’s [32] criterion whereby all scores that were one standard deviation (SD) above the mean score reflected moderately high levels of loneliness, and scores over two SDs above the mean reflected very high level of loneliness. The mean score of loneliness in the present study was 22.5 (*SD* = 5.51) and 81 participants (16.1%) were classified as having moderately high loneliness scores, and 10 participants (2%) were in the very high lonely group.

Depression and anxiety symptoms were calculated using the GAD-7 and PHQ-9 cut-off points respectively. Using the cut-off criteria for PHQ-9 recommended by Kroenke and Spitzer [29], the present results indicated that 11.9% (*n* = 60) of the participants displayed mild depression (i.e., PHQ-9 scores between 5 and 9), 5% (*n* = 25) displayed moderate depression (i.e., PHQ-9 scores between 10 and 14), and 1.4% (*n* = 7) displayed moderately severe depression (i.e., PHQ-9 scores between 15 and 19). Accordingly, 10.3% (*n* = 52) reported mild (i.e., GAD-7 scores between 5 and 9) and 4.6% (*n* = 23) reported moderate levels of anxiety (i.e., GAD-7 scores between 10 and 14). No participants reported severe depression or anxiety symptoms.

Kruskal-Wallis tests were further used to examine whether participants in the different anxiety and depression groups differed in the emotional burden of loneliness. Participants with minimal depression differed significantly in the emotional burden of loneliness compared to those with mild (*H* = -156.20, *p* < .001), moderate (*H* = -177.59, *p* < .001), and moderately severe depression scores (*H* = -218.13, *p* < .001). Furthermore, the results showed that participants with mild (*H* = -134.28, *p* < .001) and moderate anxiety levels (*H* = -199.68, *p* < .001) had significantly different scores in the emotional burden of loneliness as compared to those with minimal anxiety. Participants with mild and moderate levels of anxiety did not differ significantly in the emotional burden of loneliness.

### Sex and Age Differences in Loneliness, the Emotional Burden of Loneliness, and Mental Health Outcomes

Independent samples t-test was used to assess sex differences in the emotional burden of loneliness, and in depression and anxiety symptoms. Analysis of frequencies with Pearson’s chi-square (*χ*^2^) was also used to determine sex differences in the frequency of loneliness. The results indicated that females reported significantly higher scores in the emotional burden of loneliness (*t* = -3.24, *p* = .001) and in anxiety symptoms (*t* = -2.99, *p* = .003), than males. Non-significant sex differences were observed for depression and frequency of loneliness.

Three age groups were developed comprising young adults (18–24 years; 9.1%), middle-aged adults (25–64 years; 75.9%), and older adults (65 + years; 14.9%). Because age groups were unequally distributed, we used non-parametric tests (Kruskal-Wallis) with pairwise comparisons to determine differences in the frequency of loneliness, the emotional burden of loneliness, and depression and anxiety symptoms. The results indicated significant age differences in the frequency (*H* = 12.41, *p* = .002) and the emotional burden of loneliness (*H* = 14.09, *p* = .001). Pairwise comparisons further showed that younger adults reported significantly higher scores in the emotional burden of loneliness, compared to middle-aged (*H* = 57.64, *p* = .011) and older adults (*H* = 101.73, *p* < .001). Middle-aged adults also experienced higher emotional burden of loneliness compared to older adults (*H* = 44.09, *p* = .016). Significant age differences were also observed in symptoms of anxiety (*H* = 15.40, *p* < .001) and depression (*H* = 14.37, *p* = .001), with younger adults reporting significantly higher scores in GAD-7 and PHQ-9 than middle-aged and older adults.

### Multivariate Associations between the Emotional Burden of Loneliness and Mental Health Outcomes

Two hierarchical linear regression models were computed to respectively assess the multivariate association between the emotional burden of loneliness and symptoms of depression (Model 1) and anxiety (Model 2), after controlling for socio-demographic variables (i.e., age and sex), social network size, and loneliness frequency scores. In both models the analysis was computed in two steps, with socio-demographic variables, social isolation, and loneliness frequency added in the first step, and the emotional burden of loneliness (i.e., loneliness frequency × valence × arousal) added in the second step of the analysis. This sequence allowed us to determine the incremental predicted variance in depression and anxiety symptoms that was added after entering the emotional burden of loneliness in the model, on top of the effects of other predictors. Also, to avoid multicollinearity the scores of loneliness frequency and the emotional burden of loneliness were mean-centred.

In Model 1, the overall model predicted 28.4% (Adjusted *R*^2^) of the variance in depression symptoms (*F* = 40.78, *p* < .001, multivariate effect size *f*^2^ = 0.39). Tolerance levels were at acceptable levels (> 0.340) suggesting low multicollinearity between the predictor variables. At the first step of the analysis, depression symptoms were significantly associated only with loneliness frequency scores (*β* = 0.443, *p* < .001). The effects of age, sex, and social isolation were non-significant. At the second step of the analysis, adding the emotional burden of loneliness significantly increased predicted variance in depression symptoms by 4.7% and the effect of loneliness frequency scores was reduced but remained marginally significant (β = 0.141, p = .04). The results are summarised in Table 2.

**Table 2:** Multivariate association between the emotions burden of loneliness and depression symptoms

	Beta	β	95% CI for B	Adjusted *R*^2^
**Step 1**				23.7%
Sex	0.445	0.064	− 0.087 – 0.978	
Age	− 0.561	− 0.077	-1.137 – 0.016	
Loneliness frequency	1.568	0.443***	1.247 – 1.890	
Social isolation	− 0.032	− 0.053	− 0.087 – 0.023	
**Step 2**				28.4%
Sex	0.445	0.025	− 0.350 – 0.698	
Age	− 0.513	− 0.071	-1.072 – 0.046	
Loneliness frequency	0.500	0.141*	0.023 – 0.977	
Social isolation	− 0.033	− 0.055	− 0.086 – 0.019	
Emotional burden of loneliness	1.332	0.376***	0.881 – 1.783	

*p < .05; **p < .005; ***p < .001

Model 2 predicted 24.6% (Adjusted *R*^2^) of the variance in anxiety symptoms (*F* = 33.82, *p* < .001, multivariate effect size *f*^2^ = 0.32). Tolerance levels were at acceptable levels (> 0.304). At the first step of the analysis, anxiety symptoms were significantly associated with being female and loneliness frequency scores. The addition of the emotional burden of loneliness at the second step significantly increased the variance in anxiety symptoms by 6.2%. Importantly, the effects of sex and loneliness frequency scores turned non-significant. The results are summarised in Table 3.

**Table 3:** Multivariate association between the emotions burden of loneliness and anxiety symptoms

	Beta	β	95% CI for B	Adjusted *R*^2^
**Step 1**				18.4%
Sex	0.693	0.109*	0.188 – 1.198	
Age	− 0.528	− 0.079	-1.075 – 0.019	
Loneliness frequency	1.285	0.395***	0.980 – 1.590	
Social isolation	− 0.006	− 0.010	− 0.057 – 0.046	
**Step 2**				24.6%
Sex	0.407	0.025	− 0.350 – 0.698	
Age	− 0.478	− 0.071	-1.072 – 0.046	
Loneliness frequency	0.158	0.049	0.023 – 0.977	
Social isolation	− 0.007	− 0.013	− 0.057 – 0.043	
Emotional burden of loneliness	1.405	0.432***	0.981 – 1.830	

*p < .05; **p < .005; ***p < .001

## Discussion

The present study examined for the first time the emotional burden of loneliness, which reflects the interaction between loneliness experiences frequency, valence, and arousal, and its association with mental health outcomes. This approach is consistent with recent calls by scholars [e.g., 9] to further extend standard measures of loneliness frequency by incorporating theoretically relevant dimensions of loneliness experiences. The present study also meaningfully extends recent research that examined the emotional content of loneliness experiences but without explicitly addressing emotional valence and arousal dimensions [e.g., 12, 13]. Taken together, our findings indicate that measuring the emotional burden of loneliness provides novel insights into our understanding of loneliness experiences, and their association with mental health outcomes, in the following ways.

Firstly, two regression models respectively showed that the emotional burden of loneliness was positively associated with depression and anxiety symptoms, over and above loneliness frequency and an objective measure of social isolation. Specifically, the incremental variance added by the emotional burden of loneliness ranged from 4.7% for depression symptoms, to 6.2% for anxiety symptoms. These findings indicate that measuring the emotional burden of loneliness (i.e., valence and arousal of loneliness experiences) can significantly improve predicted variance in mental health symptoms, after controlling for the effects of loneliness frequency. This is an important finding because it shows that measuring only the frequency of loneliness provides a partial view of loneliness experiences. As Qualter et al. [9] suggested, research on loneliness may advance by exploring the dimensions of loneliness that are not captured by existing frequency measures. Furthermore, the present findings showed that the emotional burden of loneliness was significantly associated with mental health outcomes after controlling for objective measures of social isolation (i.e., social loneliness), which had a non-significant effect. This supports previous research about the theoretical, conceptual, and empirical distinction between measures of objective and subjective loneliness and further indicates that the emotional content (or burden) of loneliness may be more relevant in predicting depression and anxiety symptoms, than the size of one’s social network [10].

Another important finding is that sex differences were observed, with females reporting higher levels in the emotional burden of loneliness than males, although no sex differences were observed in loneliness frequency. This indicates that although males and females may not differ in *how often* they experience loneliness, they differ significantly in the *emotional burden* of loneliness. Moreover, there is evidence suggesting that men are less likely to report distress in standard quantitative measures of well-being than women. For example, in a qualitative study with in-depth interviews, male widowers reported depressive symptoms more often than female widows [33], but such sex differences were not observed when quantitative measures (e.g., SAD, HADS) were employed. Further research is warranted to explore potential sex differences in the emotional burden of loneliness and confirm the present findings.

Moreover, a linear trend in age differences in the emotional burden of loneliness was observed, with younger adults experiencing significantly greater emotional burden than middle and older adults. This is in contrast to research findings demonstrating a U-shaped association between age and the frequency of loneliness. Specifically, studies have shown that loneliness peaks in young adults under the age of 30, and then again in adults over the age of 80 [21, 34]. As such, the current findings offer insight into the emotional burden of loneliness across age groups, suggesting that younger people not only feel lonely more frequently than middle and older adults, but they also experience a greater emotional burden of loneliness. Future research may examine whether age differences in the emotional burden of loneliness are a function of loneliness duration (i.e., the chronicity of loneliness) [35], and whether the emotional burden of loneliness is associated with transient loneliness states induced by situational factors, such as moving away from home.

Lastly, the present findings provide early support for the nomological, construct, predictive, and incremental validity of the emotional burden of loneliness. With regards to nomological and construct validity, the bivariate associations (Table 1) indicated a linear positive relationship between the emotional burden of loneliness, loneliness frequency, and higher scores in symptoms of depression and anxiety. Accordingly, participants with differing levels of depression and anxiety, as reflected in the respective cut-off scores of PHQ and GAD, reported significantly different levels of the emotional burden of loneliness (i.e., higher burden of loneliness in people with higher depression and anxiety scores). In terms of predictive validity, the multivariate associations (Tables 2 and 3) of the emotional burden of loneliness with depression and anxiety symptoms was significant, after controlling for the effects of loneliness frequency and social isolation. Finally, evidence for incremental validity is reflected in the unique variance added by the emotional burden of loneliness in the multivariate models predicting anxiety and depression symptoms.

The present study is not free of limitations. The cross-sectional nature of the design does not allow for causal explanations of the relationship between the emotional burden of loneliness and mental health difficulties. However, several strengths should be noted. Firstly, this is the first published study that incorporates measures of valence and arousal in addition to frequency using the UCLA, a widely used self-report measure for loneliness. Secondly, gender and age differences provide novel insight into the emotional burden of loneliness across the life span. These findings have implications from interventions to alleviate loneliness particularly in younger adults, where the emotional burden of loneliness is more pronounced. Further research is needed to explore the effect of chronicity on the emotional experience of loneliness and to adopt novel approaches to therapy and practice.

## Conclusions

Incorporating dimensions of emotional valence and arousal in the assessment of loneliness frequency ratings (e.g., how often one feels lonely) allows us to identify a previously unnoticed dimension of loneliness experiences: the emotional burden of loneliness. The present study demonstrated, for the first time in the extant research, that the emotional burden of loneliness was significantly associated with depression and anxiety symptoms, over and above the effects of loneliness frequency ratings, socio-demographic variables, and objective measures of social isolation, such as social network size. Importantly, females experience significantly greater emotional burden of loneliness, even when sex differences in loneliness frequency ratings are not observed.
